# Rough Enamel

**Published:** 1885-01

**Authors:** C. E. Francis


### ROUGH ENAMEL.

BY C. E. FRANCIS, D. D. S.

A prevalent notion seems to have been long in existence that the enamel of a tooth should never be molested in any manner, and if but barely touched with either a mechanical or chemical agent its integrity is virtually destroyed.

So strongly has this idea forced its way into the mind of mankind generally, that many persons would rather risk the loss of all their masticators in the natural order of things than have their teeth cared for by a dentist. Indeed, not a few individuals refrain from the use of dentifrices, and even the tooth-brush, fearing their teeth will be ruined thereby. Not infrequently during the process of preparing a cavity for filling, or in removing stains or salivary calculus, the dentist is asked if he is not destroying the enamel of the teeth upon which he is operating, and often a word of caution or protest is uttered by the patient. Sometimes the dentist is seriously annoyed at the lack of confidence thus manifested, and at times feels somewhat hampered in his efforts to benefit those who so question the soundness of his judgment.

It is, however, refreshing to meet, as is occasionally the case, with patients sensible enough to repose trust in those whose services they seek.

It may be well to cite one or two cases of practice which will serve as a reply to the question asked by "G. B." in the August number of the INDEPENDENT PRACTITIONER, page 474.

About eighteen years ago a gentleman brought his son, a lad of twelve years, who possessed a set of teeth about as ill-looking as it is possible to imagine. The entire surface of enamel was but a tissue of serrations and pits, and, coated as they were by dark-colored accumulations, he presented a most repulsive appearance. Said the father: "They are the worst looking teeth I ever beheld, and it pains me to look at them. Can anything be done to improve them, or must they be extracted?" After due examination the following advice was imparted: "If the lad was my son I would plane down the surfaces of his teeth and polish as smoothly as possible." "Very well," was the rejoinder; "do for him as if he was your child." So with broad, sharp chisels and files, the rough surfaces were cut away, and with sticks of Superior-stone and powdered pumice were polished quite smoothly. A few pits, here and there, penetrated the dentine, and were carefully filled with gold.

The result proved a complete success, and from that time they have remained whole and clean.

Another case was that of a youth of fifteen summers, with superior incisors much eroded, their entire labial surfaces roughened by acidulated secretions. The teeth were somewhat stained and a little sensitive. The surfaces were smoothed with fine corundum wheels, and polished with corrugated rubber disks charged with finely-ground Arkansas-stone. For three years they have kept splendidly, are bright and clean, and present a far better luster than in their original or normal condition.

With the above, and many other similar experiences as a guide, I would not hesitate to cut away and polish rough enamel.

